# Rearing Temperature Influences Adult Response to Changes in Mating Status

**DOI:** 10.1371/journal.pone.0146546

**Published:** 2016-02-10

**Authors:** Erica Westerman, Antónia Monteiro

**Affiliations:** 1 Department of Ecology & Evolutionary Biology, Yale University, New Haven, Connecticut, United States of America; 2 Department of Ecology & Evolution, University of Chicago, Chicago, Illinois, United States of America; University of Arkansas, UNITED STATES

## Abstract

Rearing environment can have an impact on adult behavior, but it is less clear how rearing environment influences adult behavior plasticity. Here we explore the effect of rearing temperature on adult mating behavior plasticity in the butterfly *Bicyclus anynana*, a species that has evolved two seasonal forms in response to seasonal changes in temperature. These seasonal forms differ in both morphology and behavior. Females are the choosy sex in cohorts reared at warm temperatures (WS butterflies), and males are the choosy sex in cohorts reared at cooler temperatures (DS butterflies). Rearing temperature also influences mating benefits and costs. In DS butterflies, mated females live longer than virgin females, and mated males live shorter than virgin males. No such benefits or costs to mating are present in WS butterflies. Given that choosiness and mating costs are rearing temperature dependent in *B*. *anynana*, we hypothesized that temperature may also impact male and female incentives to remate in the event that benefits and costs of second matings are similar to those of first matings. We first examined whether lifespan was affected by number of matings. We found that two matings did not significantly increase lifespan for either WS or DS butterflies relative to single matings. However, both sexes of WS but not DS butterflies experienced decreased longevity when mated to a non-virgin relative to a virgin. We next observed pairs of WS and DS butterflies and documented changes in mating behavior in response to changes in the mating status of their partner. WS but not DS butterflies changed their mating behavior in response to the mating status of their partner. These results suggest that rearing temperature influences adult mating behavior plasticity in *B*. *anynana*. This developmentally controlled behavioral plasticity may be adaptive, as lifespan depends on the partner’s mating status in one seasonal form, but not in the other.

## Introduction

The ability to assess and respond to changes in one’s environment allows many organisms to exist successfully in a range of social scenarios and physical environments. Research suggests that both nutrition and social stimuli experienced during development influence the ability of animals to respond to variation in their adult environments (i.e. adult behavioural plasticity), presumably by influencing neurodevelopment [1–5]. Only recently have abiotic factors such as rearing temperature also begun to be examined as having an influence on adult behavioral plasticity [1]. Determining the importance of abiotic rearing environment to an adult’s ability to respond to social and environmental cues is particularly relevant now, as climate change is altering global weather patterns and the consequent rearing conditions experienced by a wide range of organisms. These changes in rearing conditions can lead to changes in emergence time, voltinism, and prey-predator synchrony in multiple species [6–11]. Adults may therefore be encountering environments that differ from those historically experienced. The ability to change behavior in response to these novel environments may facilitate an adaptive response to climate change, while a rearing-environment induced reduction in adult behavioral plasticity may be maladaptive.

Both adult social environment and rearing environment can influence mating behavior in isolation. Adult environmental conditions such as sex ratio, mating status, food availability, and perceived attractiveness of a potential mate can influence mate selectiveness and mating behavior [12–19]. Rearing temperature, juvenile nutrition, juvenile food source, and early social experience can also influence mate preference and choosiness [20–23]. However, it remains unresolved whether variations in rearing conditions also influence an individual’s ability to modify their mating behavior in response to their immediate environmental conditions, such as sex ratio, mating status, food availability, and perceived attractiveness of a potential mate.

While it is unclear whether abiotic rearing conditions influence mating behavior plasticity, abiotic environmental cues, such as photoperiod, are associated with potentially adaptive seasonal changes in behavior for many species of animals. In species where photoperiod is predictive of mating season, portions of the brain associated with courtship and mate acquisition increase in size (birds) and responsiveness to stimuli (mammals) in response to changes in photoperiod (reviewed in [24, 25]). The hippocampus of food caching birds also undergoes seasonal changes in size which correspond to seasonal changes in spatial memory necessary for remembering food cache locations [1]. Seasonal cues influence adaptive behaviors in insects as well, though these seasonal changes in behavior are not as well understood [26] (but see [27–29] for neurophysiology associated with migration in the monarch butterfly, *Danaus plexippus*). Adult and larval diapause are induced by seasonal cues such as photoperiod and temperature [26], and many insects exhibit seasonal migratory behavior that is adaptive and hypothesized to be induced by a seasonal cue [30, 31]. However, the effect that abiotic developmental cues associated with season and climate change, such as temperature, have on adult behavioral plasticity remains ambiguous.

The abiotic developmental environment may have particularly strong effects on adult behavioral plasticity in species that are multivoltine and either undergo seasonal reproductive diapause or are seasonally polyphenic, i.e., that exhibit different adult forms in the different seasons of the year, traits which are common in insects (reviewed in [26]). In these species, different generations experience different environments and selection pressures [32]. Rearing conditions are generally predictive of adult conditions, and are used as cues for development of the different adult forms in many of these systems. Morphological traits associated with seasonal polyphenisms and reproductive diapause are relatively easy to quantify, and have been well documented in a variety of systems [33, 34]. However, the environmental variation that selects for this dramatic seasonal variation in morphology may also select for variation in behavior, as predation risk, food availability, and habitat may also be seasonal. We expect that rearing conditions may influence morphology, reproductive costs, and behavior in these systems. Rearing conditions may have a large effect on reproductive costs and behavior in insects because males of multiple species of insects transfer nutrients or nuptial gifts along with their spermatophores during mating, and these nuptial gifts can be a major source of nutrients for females [35–37]. These nuptial gifts may be particularly important in environments where other nutritional resources are rare, such as dry or cold seasons, or high-density environments [21, 38, 39]. In addition to varying with rearing conditions, nuptial gift quality may change throughout a male’s lifetime, or in response to perceived female quality [35, 40, 41]. In systems where resource availability and the consequent value of nuptial gifts fluctuate, female and male choosiness has been found to fluctuate in tandem with this fluctuation in nuptial gift benefits to females and costs to males [21, 36, 38, 42]. Thus, if the direct benefits or costs of mating are dependent on rearing conditions, one might expect adult mating behavior plasticity to also be rearing condition dependent. Here we examine whether rearing temperature alters either the benefits of mating multiply or the costs of mating with non-virgins, and consequently influences adult behavior in a species with seasonal alternative adult forms, the butterfly *Bicyclus anynana*.

**Fig 1:**
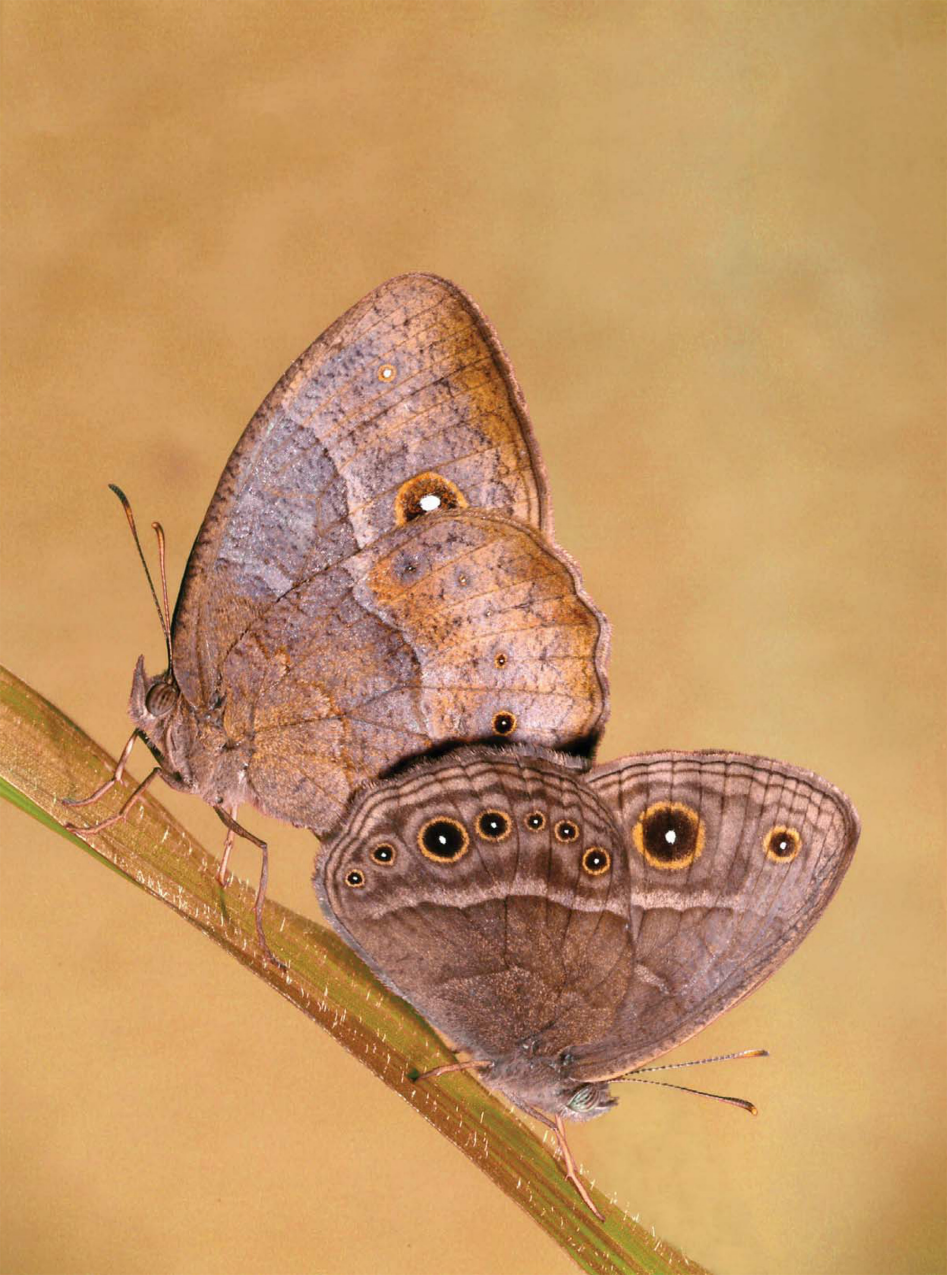
Variation in wing patterning of WS and DS *B*. *anynana* butterflies. Here we show a DS female and WS male mating in the laboratory. The DS female is the upper left of the two mating butterflies, while the WS male is the lower right butterfly. DS butterflies have smaller ventral eyespots than WS butterflies, and a more mottled brown patterning on the ventral surfaces of their wings. While wing size is sexually dimorphic in this species, the seasonal variation in eyespot size and general ventral wing surface coloration is sexually monomorphic.

*Bicyclus anynana* is a subtropical African butterfly whose natural habitat fluctuates annually from a wet and warm environment to a dry and cool environment. This seasonal shift corresponds to a shift from high to low foliage and larval food quality and availability, and potentially a shift in predator identity [43–46]. *B*. *anynana* butterflies are present in both seasons, but in two discrete forms, a wet season (WS), and a dry season (DS) form [44]. The two seasonal forms differ in wing patterning, with the WS form having larger eyespots on the ventral surfaces of their fore-and hind-wings relative to DS individuals, as well as other more subtle differences in shading (Fig 1). The two seasonal forms also exhibit different sexual behaviours: DS males are selective and engage in less courtship behavior than WS males [21, 47], whereas DS females engage in more courtship behavior and are less selective than WS females [21]. This change in male and female courtship behavior is independent of whether males or females are interacting with butterflies of the opposite seasonal form: WS males court more than DS males when paired with both DS and WS females, and DS females court more than WS females when paired with both DS and WS males [21]. Variation in rearing temperature is sufficient to induce this seasonal variation in wing patterning and courtship behavior, with butterflies exhibiting WS wing patterning and courtship behavior when reared at 27°C, and exhibiting DS wing patterning and courtship behaviour when reared at 17°C, independent of adult culturing temperature [47, 48]. This seasonal variation in behavior corresponds to seasonal variation in the costs and benefits of first copulation to males and females: virgin DS males produce resource rich spermatophores, which lead to increased longevity of DS females, but reduce their own longevity after mating [21]. A first mating, on the other hand, does not change the longevity of WS females and males relative to unmated individuals [21]. The direct benefit DS females experience when mating with a DS male is independent of female rearing temperature, as is the direct cost of mating to DS males: WS females mated to DS males experience increased longevity relative to virgin WS females, and DS males mated to WS females experience decreased longevity relative to virgin DS males [21]. It is unknown whether these rearing temperature dependent mating benefits and costs extend to second matings for either sex.

Given the large direct benefit of mating to DS females, and the lack of direct benefits of mating to WS females, one might expect DS females to seek out multiple matings, as is observed in other insect species where females experience large benefits from mating [49, 50], and WS females to avoid remating, as occurs in other butterfly species where females do not receive large direct benefits of mating [51]. Given the large cost of a first spermatophore to DS males, one might expect them to invest less in a second spermatophore, as occurs in other species of butterflies [52], and for the difference in quality of first and second spermatophores to be greater in DS males than in WS males. This predicted temperature-dependent plasticity in spermatophore quality may lead DS individuals to behave differently when in the presence of a mated or a virgin individual of the opposite sex, while no such behavioural plasticity is expected of WS individuals. It may also lead to WS females but not DS females becoming more reluctant to mate after a first mating. We tested these hypotheses by examining the direct benefits and costs of mating multiply on lifespan of DS and WS male and female butterflies. As likelihood to remate is also dependent on the willingness of an individual of the opposite sex to mate with a non-virgin, we also examined the benefits and costs of mating with virgins versus non-virgins on DS and WS male and female butterfly lifespan. We predict that DS females will experience greater benefits from mating multiply than WS females. We also predict that DS males will experience greater costs from mating multiply than WS males.

If there is a rearing environment effect on the reproductive benefits and costs of mating with a non-virgin, *B*. *anynana* of different seasonal forms may also experience variation in their incentive to remate. DS females but not WS females incur a direct benefit of mating with virgin males of their seasonal form [21]. Therefore, we predict that DS females will be more likely to mate multiply, and less likely to change their intersexual behavior after mating than WS females. Virgin DS males but not virgin WS males incur a direct cost of mating, and DS males are more selective than WS males [21]. Therefore, we predict DS males will be more sensitive to, and more likely to change their sexual behaviour in response to the mating status of their potential partner.

To test these hypotheses, we manipulated rearing temperature and mating status of males and females, and measured the lifespan of male and female individuals after a first mating, after a second mating, and after mating with virgins and non-virgins. We then measured the effect of rearing temperature and male and female mating status on male-female premating interactions and copulation duration. This full factorial design allows us to assess whether any seasonal variation we might observe in mating behavior is due to seasonal variation in costs of remating, costs of mating with non-virgin individuals, or both. Seasonal variation observed in either the behaviour of mated females or the response of males to previously mated females could be the result of female developmental environment, or the effect of developmental environment on first mate’s spermatophore content. To explore whether rearing temperature had an effect on behavioral plasticity indirectly via male spermatophore contents, we compared the behavior of females previously mated to either DS or WS males in their interactions with virgin males.

## Materials and Methods

### Study Animals

*Bicyclus anynana* is a subtropical African butterfly that has been in laboratory culture since 1988. A colony was established in New Haven, CT in 2006 from hundreds of eggs collected from a laboratory colony in Leiden, The Netherlands (originally established from 80 gravid females collected in Malawi in 1988). Laboratory populations contain similar single-nucleotide polymorphism (SNP) frequencies to those of current natural populations [53, 54], suggesting that laboratory breeding practices have maintained levels of genetic diversity similar to those found in natural populations.

Despite the absence of a description of *B*. *anynana* mating displays in natural conditions, observations made in large greenhouse spaces, climate rooms, and smaller rearing cages suggest that WS *B*. *anynana* males patrol to find females, and perching WS females accept or reject males that approach them [21, 55–57]. The stereotypic courtship behavior exhibited by males of these multiple breeding populations suggests that the butterfly behavior observed in the experimental conditions described below is not specific to this particular breeding population, and likely contains a significant representative range of male and female behavioral responses to mating opportunity.

### Animal Husbandry

For the experiments described below, all butterflies were reared in Environair® walk-in climate controlled chambers with 12:12 light: dark cycle, 80% humidity, and either at 27°C, inducing the WS form, or at 17°C, inducing the DS form. Light intensity was gradually increased or decreased (over a period of 45 min) in the climate-controlled chambers to approximate the natural change in light levels that occurs at dawn and dusk. Eggs were laid on young corn (*Zea mays*) plants in breeding cages (cylindrical hanging net cages, 30cm diameter X 40 cm height) containing ~50 butterflies of mixed sex. Plants with eggs were removed from breeding cages every 2–3 days and transferred to aluminium rearing cages (17” x 17” x 24”) containing ~12 corn plants for larvae consumption. Larval cages were cleaned and new plants added every two days, and sometimes more frequently when plant consumption was high. All caterpillars were fed on young corn plants for all five larval instar stages. Pupae were removed from larval cages and placed in cylindrical hanging net cages until emergence. Previous studies have determined that rearing *B*. *anynana* at these conditions consistently produces butterflies with WS (when reared at 27°C) or DS (when reared at 17°C) wing patterning [58]. Adults were separated by sex at time of emergence, and maintained in age specific small hanging cages (6” x 6” x 8”) until day 3 after emergence: the start of each experiment. All cages were visually isolated from each other by cage-sized pieces of opaque construction paper. All butterflies were given wetted cotton and banana daily, unless otherwise specified below. Development time was ~4 weeks from 1^st^ instar to adult for butterflies reared at 27°C, and ~8 weeks for butterflies reared at 17°C. All butterflies reared at 27°C emerged with WS wing patterning, and all butterflies reared at 17°C emerged with DS wing patterning.

Butterflies were only removed from the climate-controlled chambers for the fifteen minutes prior to the one-hour behavioral assays, which were conducted in a separate, climate-controlled viewing area. In order to differentiate between the effect of developmental rearing temperature and adult ambient temperature on adult behavioral plasticity [47], we observed all butterflies at a common temperature of 25–28°C. While this observation temperature is more similar to the WS rearing temperature than to the DS rearing temperature, it is the temperature used for comparative behavioral assays in previous studies on developmental plasticity and behavioral plasticity in *B*. *anynana* [18, 21]. To produce data comparable to that of these earlier studies, we used the same temperature conditions, as well as the same light conditions and observation area [18, 21]. Previous work found that virgin male butterflies reared at different temperatures maintain rearing temperature dependent rates of courting behaviour when observed in large social groups, independent of adult culturing temperature (27°C or 17°C) [47]. These findings suggest that observing the butterflies at a common temperature should not mask rearing temperature effects on adult courting behavior. In order to limit observer bias, observational cages were arbitrarily numbered, and the treatment that corresponded to each numbered cage was recorded in a separate notebook from that used by the observer to record behavioral data.

### Lifespan Assay

We recorded lifespan for 10 individuals from each seasonal form and sex in the following five mating status categories: 1) virgins; 2) individuals mated once to a virgin; 3) individuals mated once to a non-virgin: 4) individuals mated twice to virgins; and 5) individuals mated twice, first to a virgin and then to a non-virgin. All mates were of the same seasonal form as the test individual. After being reared to pupation in respective 27°C and 17°C climate controlled chambers, WS and DS butterflies (still in their respective 27°C and 17°C climate controlled chambers) were segregated by sex on day of emergence (day one) placed in age specific, visually isolated small (6” x 6” x 8”) hanging cages, and given banana and water until day three. On day three individual butterflies were moved to visually isolated small hanging cages within the climate controlled chambers containing cotton soaked in water but no banana, and either completely isolated or placed in a cage with a single butterfly of the opposite sex. The following day (day four) individuals that had been paired were checked for mating (mating was determined by the use of florescent rodent tracker powder, as described in [55] and [59]). If mating had occurred, the original individual was paired with a second individual of the opposite sex (virgin or non-virgin, depending on treatment) or was isolated depending on treatment. On day five paired individuals were checked for mating, and all individuals were then isolated in independent cages containing cotton soaked in water and checked daily until demise. The pairs (of both first and second matings) that did not mate within 24 hrs of being placed together in a cage were not used for this study in order to maintain researcher-manipulated and not self-selected treatments. Cotton was rewetted every two days to insure that death was not due to dehydration. This experimental design has been previously used as a method to limit demise due to dehydration in measurements of lifespan of *B*. *anynana* butterflies [21]. All life span data were obtained from individuals that were maintained in their respective climate controlled chambers so that we could determine how mating influenced life span at ambient temperatures similar to those that would be experienced in nature. This was a different set-up from the one in Prudic et al. (2011), where all animals were kept at 25–27°C. We measured the effect of mating on lifespan instead of egg production to remove any confounding effects of cryptic female choice or maternal effects on our estimation of the direct benefits of mating.

### Behavioral Assay

To determine whether rearing environment influences plasticity in *B*. *anynana* mating behavior, we observed pairs of WS-WS and DS-DS butterflies in four different social scenarios: 1) both virgin; 2) both non-virgin; 3) male non-virgin and the female virgin; or 4) male virgin and the female non-virgin. N = 15 pairs per treatment per seasonal form. We conducted all our experiments in within seasonal form pairs because we were interested in the interactive effect of rearing temperature and mating status on biologically relevant intersexual interactions.

Butterflies were segregated by sex on the morning of emergence, day one, and given banana and water until day three. On day three, butterflies were randomly either designated as virgins and isolated in their own small (6” x 6” x 8”) hanging cage, or designated as non-virgins, and put in a small hanging cage with one butterfly of the opposite sex. All cages contained banana and water for nourishment, and were visually isolated from all other cages by pieces of opaque construction paper. The following morning (day four) thirty minutes to an hour after lights on (between 6:50am and 7:30am) all pairs were examined to insure mating had occurred. All butterflies that had been designated as non-virgins and placed in pairs but did not mate were excluded from the study. Individuals were then placed in pairs with a previously unseen butterfly of the opposite sex in one of the five mating status social pairings described above in cylindrical hanging net cages (30cm diameter X 40 cm height). These cages were then removed from the climate controlled chambers and visually isolated from all other cages in an observational area, with full spectrum sun lamps and East facing windows. Many hours of behavioral observations under these conditions have led us to conclude that mixed sex pairs are capable of engaging in prolonged bouts of aerial interactions that influence mating outcome in laboratory conditions [18, 59, 60].

We conducted an additional treatment for each seasonal form to assess the impact of spermatophore type on female receptivity or attractiveness: we paired a female butterfly, non-virgin, but previously mated to a male of the alternative seasonal form, with a virgin male butterfly of her own seasonal form for behavioral observation. For these additional treatments, males were transferred from their rearing temperature chamber on day three to that of the other rearing temperature and placed with a female who had been reared within that chamber (WS males transferred to 17°C chamber and paired with DS females, and DS males transferred to 27°C chamber and paired with WS females). If spermatophore type and not the rearing condition of the interacting pair was responsible for any observed effects of rearing environment on behavioral plasticity, we would expect WS pairs containing a female previously mated to a DS male to behave like DS pairs containing a female previously mated to a DS male, and DS pairs containing a female previously mated to a WS male to behave like WS pairs containing a female previously mated to a WS male.

All interactions and behaviors were observed (ie continuous sampling) for 1hr following a 15 minute acclimation period immediately after being placed in the observational area. Observations began 1–2.75 hours after lights on (between 7 and 9:45am), May -July, 2011, and were conducted by a single observer.

We recorded number of incidents of the following behaviors: 1) flight; 2) wing flutter (categorized here as a single flutter of the wings made by a stationary butterfly sitting on the side of its cage; 3) walk (walking along the surface of the cage); 4) a previously described stereotypic courtship behaviour [57], and 5) copulation. Courtship consists of a series of ritualized behaviors, involving *localization* (locating and approaching a female), *orientation* (male orients his body perpendicular to the posterior of the female), *flickering* (rapid fluttering of wings), a *thrust* (male touches female’s wings with his head), and *attempt* (curling of the abdomen) [57]. To calculate rate of pre-copulatory behaviour per minute, we divided the number of incidents of each behavior that occurred prior to copulation for each individual by duration (in minutes) of trial that occurred before copulation began. We also recorded latency to copulation: minutes after start of observational period at which copulation began; and duration of copulation: minutes that the male and female butterflies were joined in copula.

### Statistical analyses

Variation in life span was examined using a full factorial parametric regression model with Weibull distribution to account for the nonparametric nature of survival data, with sex, seasonal form, partner’s mating status, and number of mates as possible parameters.

Behavioral data were tested for equal variance using a Kolmogorov Smirnov two-sample test. Datasets not conforming to homoscedasticity were analysed with non-parametric tests. A principle components analysis was conducted on rate of pre-copulatory behavior and PC1, PC2, and PC3 were used as proxies for pre-mating rates of behavior.

Rearing temperature and mating status dependent variation in duration of copulation was examined using a series of GLM models ranging from single factor to full factorial, to find the model of best fit, with rearing temperature, male mating status, and female mating status as parameters in the models. Variation in latency to copulation and likelihood to mate were examined using full factorial GLMs with rearing temperature, male, and female mating status as factors in the models.

Since we were interested in whether rearing temperature had an effect on mating status related behavioral plasticity, we compared mated WS females to virgin WS females, and mated DS females to virgin DS females, and assessed whether there was an effect of mating status on females of one seasonal form but not the other using generalized linear models (GLM). Variation in adult activity levels (or Principle Components of correlated activities) and frequency of and latency to copulation were compared using full factorial GLMs containing all data for females, and using mating status, seasonal form (rearing temperature), and an interaction between mating status and seasonal form as variables. Since our females were also interacting with males that were mated or virgin, male mating status was also included as a factor in our model. We used this generalized linear model to determine which of our independent variables, seasonal form, female mating status, and male mating status, have a significant effect on female behavior in mixed sex pairs. We included the interaction factor female mating status* season to test for a significant effect of rearing temperature on mating status dependent plasticity. We used the interaction factor male mating status*season to test for a significant effect of rearing temperature on behavioral response to a potential partners mating status. We conducted similar GLMs for male behaviors (Principle Components of correlated activities, and frequency of and latency to copulation) to determine whether there was a significant effect of rearing temperature on mating status dependent plasticity or behavioral response to a potential partner’s mating status in males.

To test whether any variation in female behaviour 24hrs post-copulation was the result of rearing temperature dependent variation in male spermatophore contents transferred to the females, behaviors of females paired with virgin males of their own seasonal form were compared with behaviors of females paired with males of the opposite seasonal form using ANOVA. Separate analyses were done for each female seasonal form.

Pre-mating rates of behavior (excluding copulation) were compared across seasonal forms using Kruskal-Wallis pair-wise comparisons of each social interaction by sex. All statistical analyses were conducted in the JMP 10.0 statistical package by SASS.

### Ethics statement

All *B*. *anynana* butterflies were maintained in laboratory conditions as specified by U.S. Department of Agriculture permit P526P-12-04897. After behavioral assays, butterflies were maintained in breeding colony cages in a climate-controlled, walk-in chamber maintained at wet or dry season conditions with ample food and water until natural death. All butterflies used in lifespan studies were maintained climate-controlled, walk-in chambers maintained at wet or dry season conditions in cages with ample water until death, as previously described in [21].

## Results

### Mating with a non-virgin reduces lifespan of WS but not of DS individuals

**Fig 2:**
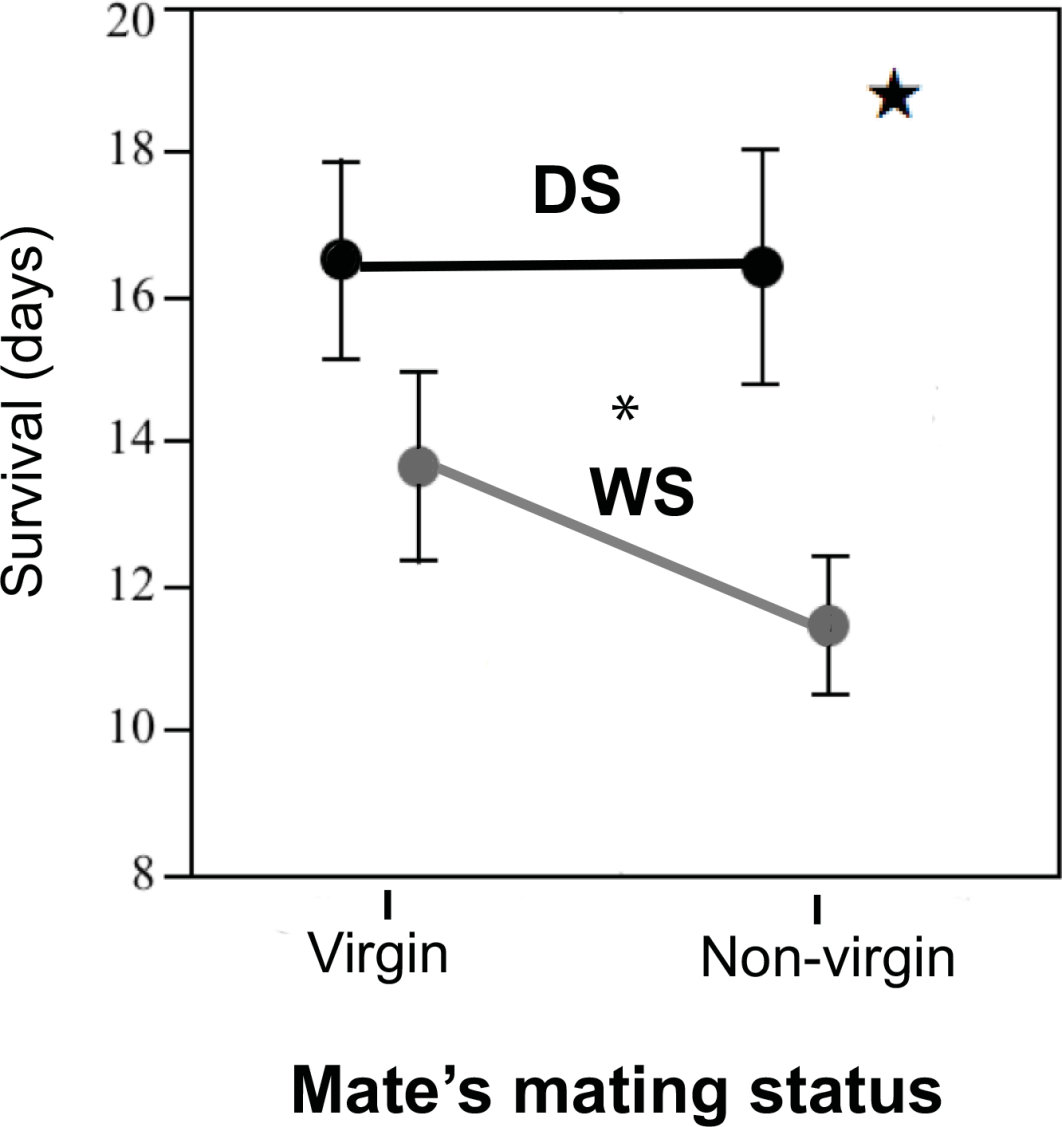
WS, but not DS individuals incur a longevity cost when mating with non-virgins instead of virgins. Visualization of the interactive effect of seasonal form and partner’s mating status on life span (data for males and females combined). Star signifies p = 0.021 for interaction between seasonal form and partner’s mating status. Asterisk (*) indicates p<0.01 for mating status of mate.

Lifespan variation was dependent on sex, rearing temperature, partner’s mating status, and the interactions of rearing temperature * partner’s mating status, and number of mating events * partner’s mating status. These five parameters were significant parameters in the parametric regression model of lifespan (AICc = 1323.03, BIC = 1390.22, whole model *χ*^*2*^ = 95.72, *P* <0.0001; sex: *χ*_*1*_^*2*^ = 27.93, *P* <0.0001; rearing temperature: *χ*_*1*_^*2*^ = 48.25, *P* <0.0001; partner’s mating status: *χ*_*1*_^*2*^ = 15.50, *P* = 0.0004; rearing temperature*partner’s mating status: *χ*_*3*_^*2*^ = 7.71, *P* = 0.021; number of mating events*partner’s mating status: *χ*_*3*_^*2*^ = 14.08, *P* = 0.0002; S1 Table). Females lived longer than males. Individuals reared and maintained at low (DS) temperatures lived longer than those reared and maintained at high (WS) temperatures. Individuals mated to two virgins lived longer than those mated to a virgin and a non-virgin. Both males and females reared at high (WS) temperature, but not those reared at low temperature (DS), incurred a longevity cost when mated to non-virgins instead of virgins (Fig 2). While there may be a trend towards an interaction effect of rearing temperature and mating status of second mate on lifespan, we did not detect a significant interactive effect between these two factors in our study. The butterfly’s own mating status (virgin or non-virgin) was not a significant factor in predicting variation in lifespan, nor was number of matings.

### Principal component analysis of behavior

Rates of behavior for males and females (pre-copula) were primarily captured in the first three principle components (PC). PC1 contained fairly equal loadings of rate of flutters, flights, walks, and courting for males, and fairly equal loadings of rate of flutters, flights, and walks for females, and explained 66.76% and 53.95% of the variance in male and female behavior, respectively (Table 1). PC1 was therefore used as a proxy for general activity level for each sex in further analyses. While rate of courting was highly correlated with rate of flying, fluttering, and walking in males, female courting was rarely observed, with only 23/148 females (9/74 WS females, 14/74 DS females) exhibiting any stereotypical courtship behavior. In addition, female rate of courting was not highly correlated with female rate of flying (R = 0.17), fluttering (R = 0.124), or walking (R = 0.210), and was the primary factor in PC2 for the female PCA analysis, which explained 23.24% of the variance in female behavior. Male PC2 was composed predominantly of positive courting and negative walks, and explained 20.86% of the variance in behavior. Female PC3, which was composed predominantly of positive flying and negative walking for females explained an additional 17.45% of the behavioral variance, while male PC3 was composed of predominantly positive courting and walking, and negative flying, and explained an additional 8.17% of the behavioral variance. Within pair male and female activity levels (PC1, PC2, and PC3) were correlated for some, but not all, of the different treatments (S2 Table).

**Table 1:** Factor loadings for Principle Components analysis of behaviors for males and females.

	Male			Female		
Behavior	PC 1	PC 2	PC 3	PC 1	PC 2	PC 3
**Flutters/min**	0.569	-0.152	-0.176	0.620	-0.167	-0.197
**Flight/min**	0.547	0.151	-0.646	0.463	-0.214	0.832
**Walk/min**	0.446	-0.665	0.492	0.583	-0.050	-0.504
**Court/min**	0.420	0.716	0.557	0.247	0.960	0.121
**Cumulative % variation explained**	66.76	87.62	95.79	53.96	77.19	94.65

### Copulation rates are developmentally plastic and dependent on female mating status

**Fig 3:**
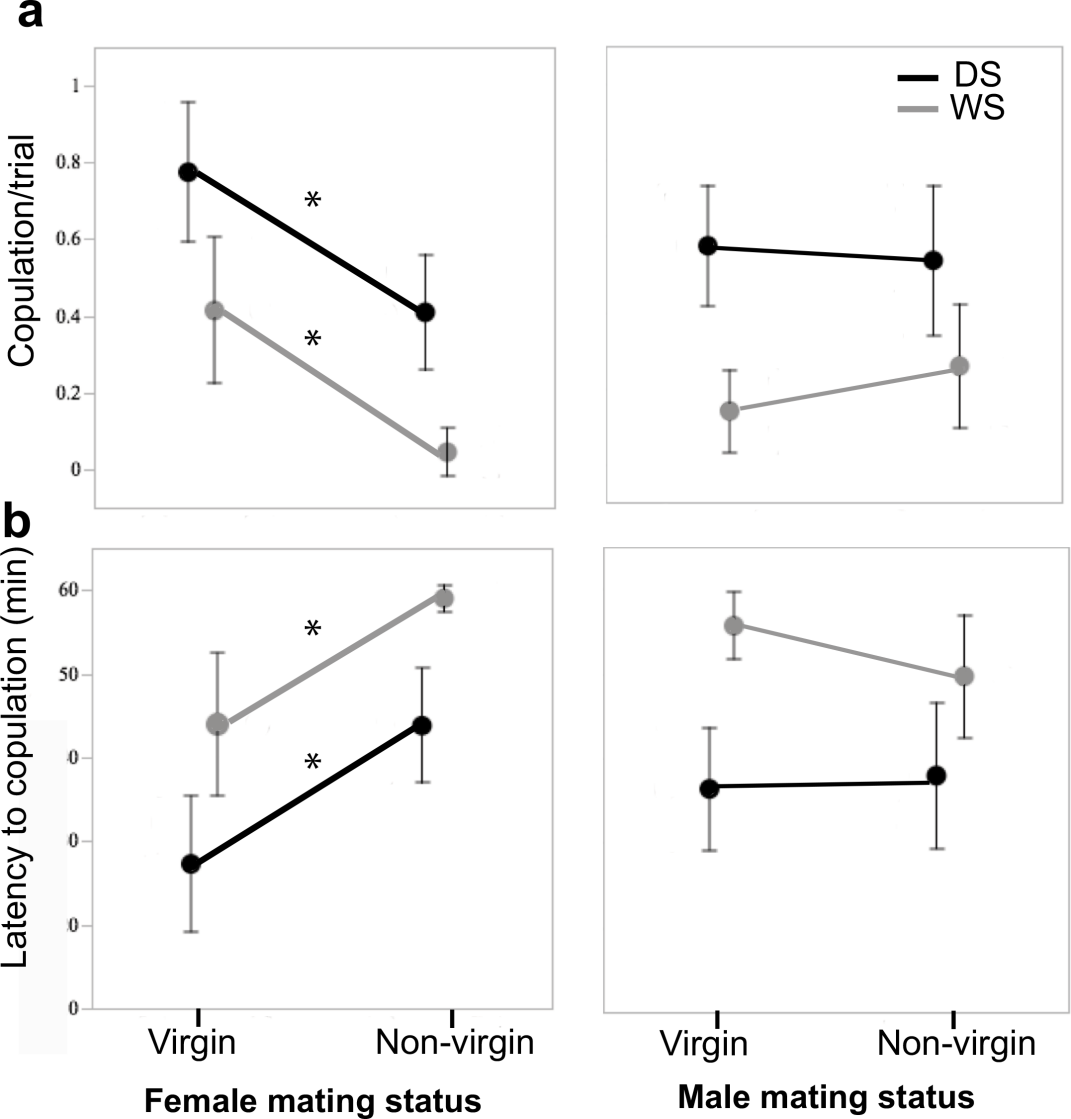
Seasonal form and mating status influence frequency and latency to copulation. Effect of seasonal form and male and female mating status on **a)** frequency of copulation, and **b)** latency to copulation for pairs of WS and DS butterflies. Panels on the left illustrate the effect of female mating status on frequency of and latency to copulation in the mixed sex pairs. Panels on the right illustrate the effect of male mating status on frequency of and latency to copulation. Seasonal form and female mating status were the two significant parameters in GLMs for frequency of copulation and latency to copulation. Non-virgin females have lower copulation frequency and higher latency to copulation than virgin females, but this effect is not present in males. DS forms of each sex display higher copulation frequency and lower latency to copulation than WS forms. Asterisk (*) indicates p<0.01.

Variation in both likelihoods of copulation and latency to copulation were associated with rearing temperature and female mating status during the one-hour observational period for all the pooled treatments. There was no effect of male mating status on either likelihood of copulation or latency to copulation. Rearing temperature and female mating status were the two statistically significant parameters in the full factorial GLM analyses for likelihood of copulation and latency to copulation (Likelihood of copulation: AICc = 165.66, whole model test *χ*^*2*^ = 45.60, *P*<0.0001, season *χ*_*1*_^*2*^ = 23.44, *P*<0.0001, female mating status *χ*_*1*_^*2*^ = 22.27, *P*<0.0001, Fig 3A; Latency to copulation: AICc = 1311.80, whole model test *χ*_*1*_^*2*^ = 48.22, *P*<0.0001, season *χ*_*1*_^*2*^ = 23.68, *P*<0.0001, female mating status *χ*_*1*_^*2*^ = 22.85, *P*<0.0001, Fig 3B, S3 Table). DS forms (both males and females) copulated more (55.26% vs 19.74%) and had lower latency to copulation (36.4 min vs 52.55 min) than WS forms. Virgin females (both WS and DS) copulated more (58.1% vs 23.3%) and had lower latency to copulation (35.1 min vs 50.9 min) than previously mated females.

### Behavioral plasticity in male copulation duration is developmentally plastic

**Fig 4:**
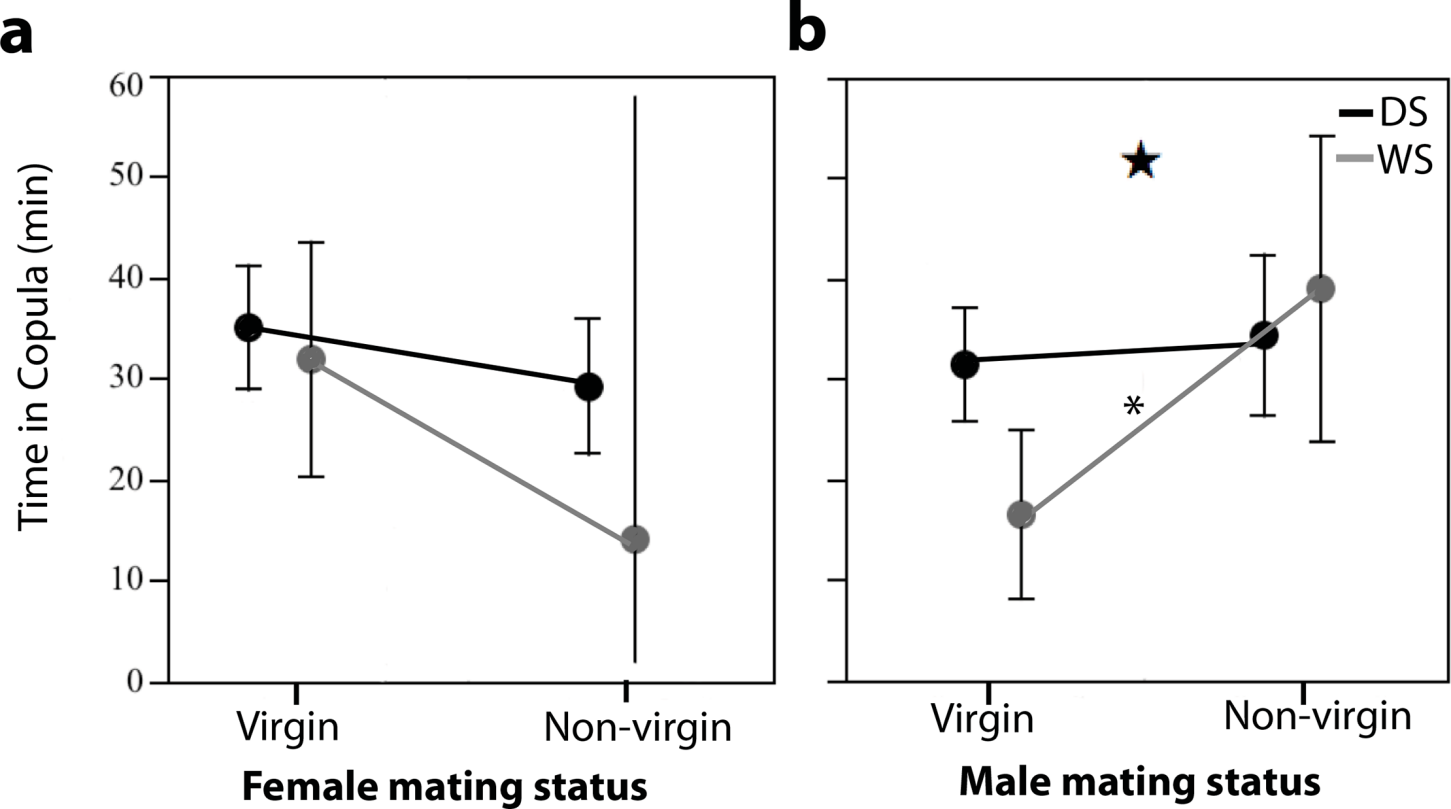
Non-virgin WS males, but not DS males, increase their time in copula relative to that of virgin males. Visual representation of the main factors in the GLM of time in copula. **a)** Effect of seasonal form and female mating status on copulation duration (female mating status does not alter time in copula for either seasonal form). **b)** Effect of seasonal form and male mating status on copulation duration. Star signifies p = 0.025 for interaction between mating status and rearing environment. Asterisk (*) indicates p<0.01.

Copulation duration was influenced by male mating status and an interaction of rearing environment (seasonal form) and male mating status. There was no effect of female mating status on copulation duration. WS mated males were in copula longer than WS virgin males (39.0 min vs 18.1 min), while DS mated males were in copula for the same amount of time as DS virgin males (36.0 vs 31.4 min) (Fig 4) (GLM, model of best fit contained rearing environment, male mating status, and male mating status*rearing environment, with significant effects attributed to the last two factors: AICc = 437.45, whole model test *χ*^*2*^ = 9.60, *P* = 0.022, male mating status *χ*_*1*_^*2*^ = 8.15, *P* = 0.004, male mating status*rearing environment *χ*_*3*_^*2*^ = 94.99, *P* = 0.025, S4 Table).

### Behavioral responses to mating status are sex specific and dependent on rearing environment

**Fig 5:**
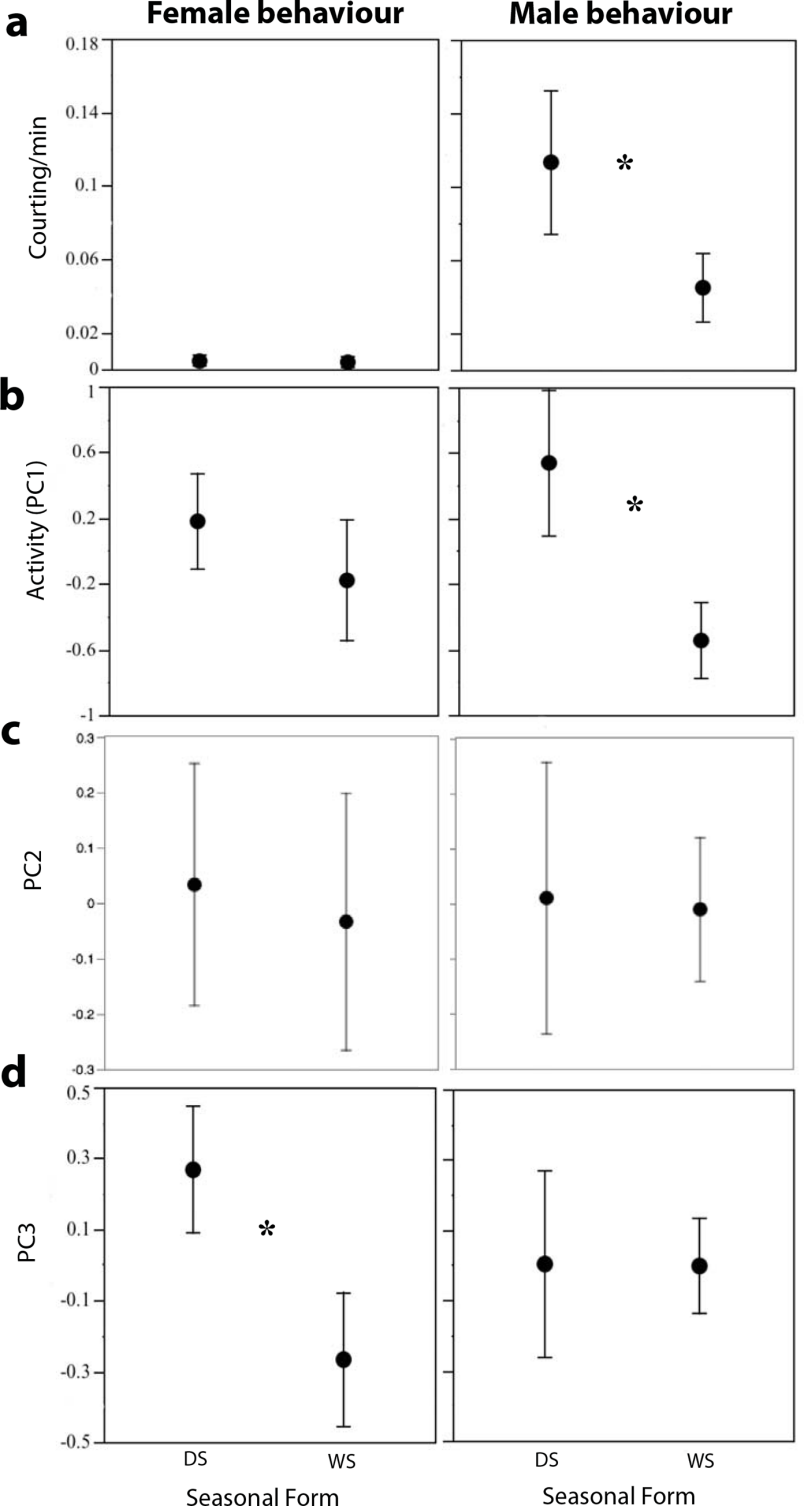
DS males courted more and were more active than WS males, whereas DS females flew more but walked less than WS females. Effect of rearing conditions on male and female **a)** courting rates; **b)** activity levels (PC 1); **c)** PC2; and d) PC 3. Asterisk (*) indicates p<0.01.

Variation in the composite behavior variables (PC1, PC2, PC3) or courtship rates for either males or females of both forms analysed together was explained only by rearing environment (seasonal form), and not by male or female mating status when these three factors were included in GLMs for these three variables (S5 Table). This is potentially due to the relatively large effect of rearing environment on activity levels (described in detail below, and illustrated in Fig 5) as compared to the effect of mating status on activity levels in individuals reared in the same conditions.

To detect subtler effects of mating status on male and female activity levels potentially masked by the large effect of seasonal form on activity levels, we analysed the behavioral data for each seasonal form and sex separately, using a full factorial GLM with male mating status, female mating status, and their interaction, as the parameters in the model. Mating status had a significant effect on activity levels for both males and females reared in WS conditions (S6 Table), but not for those reared in DS conditions (S7 Table).

Some of the effects of mating observed in WS individuals were complex. In the presence of a mated WS female, mated WS males displayed reduced courting and increased walking (i.e., lower PC2 scores) relative to virgin WS males. However, mated and virgin WS males exhibited similar PC2 scores in the presence of a virgin female (full factorial GLM containing female mating status, male mating status, AICc = 131.33, whole model test *χ*^*2*^ = 9.67, *P* = 0.021, female mating status*male mating status *χ*_*3*_^*2*^ = 7.68, *P* = 0.006). WS females exhibited higher PC2 scores (i.e., higher courting) when paired with non-virgin males than with virgin males (AICc = 201.897, whole model test *χ*^*2*^ = 12.003, *P* = 0.007, female mating status *χ*_*1*_^*2*^ = 4.27, *P* = 0.039, male mating status *χ*_*1*_^*2*^ = 6.347, *P* = 0.012). WS mated females were also less likely to court than WS virgin females (AICc = -430.59, whole model test *χ*^*2*^ = 8.86, *P* = 0.031, female mating status *χ*_*1*_^*2*^ = 5.39, *P* = 0.020).

### Spermatophore type does not alter female receptivity or attractiveness

There was no significant effect of spermatophore type (from WS or DS males) on the behavior of mated WS and DS females, or on the behavior of WS and DS males around mated WS and DS females (Table 2). Nor was there an effect of spermatophore type on likelihood to copulate, latency to copulation or copulation duration for individuals from either rearing environment (Table 2). We consequently pooled these data in our analyses of rearing-temperature-dependent behavioral plasticity.

**Table 2:** No effect of spermatophore type on female behavior or male response to mated females, regardless of female rearing environment.

	WS virgin vs non-virgin females		DS virgin vs non-virgin females	
Behavioral variable	F	p-value	F	p-value
**Female PC 1**	2.191	0.146	0.0004	0.985
**Female PC 2**	1.845	0.182	0.555	0.460
**Female PC 3**	0.921	0.343	2.181	0.147
**Likelihood to copulate**	2.327	0.139	1.189	0.285
**Latency to copulation**	1.470	0.236	0.236	0.631
**Duration of copulation**	NA	NA	0.01	0.923
**Male PC 1**	0.725	0.402	0.189	0.667
**Male PC 2**	0.004	0.951	1.564	0.221
**Male PC 3**	1.776	0.190	0.062	0.804

Results from ANOVAs comparing premating behavior, likelihood of copulation, latency to copulation, and duration of copulation for WS and DS pairs containing non-virgin females previously mated to either WS or DS males.

### Sex-specific patterns of developmental plasticity

DS males were more active than their WS counter parts for all the pooled treatments when observed at a common temperature shortly after dawn (Fig 5) (PC1 Kruskal-Wallis Test *χ*_*1*_^*2*^ = 26.396, *P*<0.0001). DS males also courted more than WS males (courting Kruskal-Wallis Test *χ*_*1*_^*2*^ = 14.999, *P* = 0.0001). DS females were not more active in general than their WS counterparts (PC1 Kruskal-Wallis Test *P*>0.05). However, DS females flew more and walked less (had higher PC3 scores) than WS females (PC3 Kruskal-Wallis Test *χ*_*1*_^*2*^ = 14.824, *P*<0.0001).

### Results Summary

WS males and females, but not DS males and females, incurred a longevity cost when they mated with a non-virgin instead of with a virgin. Rearing temperature also influenced male and female behavioral responses to mating status, as well as both male and female adult activity levels. WS males and females changed their behavior in response to mating status (either their own or that of their partner) while DS males and females did not. In general, DS individuals copulated more readily than WS individuals, and DS males were more active than WS males, independent of mating status. The observed developmental plasticity in adult behavioral response to mating status was not the result of temperature induced developmental plasticity in male first spermatophore content.

## Discussion

We predicted that DS but not WS females would benefit from mating multiply, and that DS males would experience greater costs from mating multiply than WS males, due to resources allocated to spermatophores in DS but not WS males. We also predicted that DS and not WS males would be reluctant to mate with non-virgin females, and that WS but not DS non-virgin females would be reluctant to remate. However, our results did not support these predictions. We found that neither DS nor WS females benefit greatly from mating multiply, nor do DS or WS males suffer greatly from mating multiply. Instead, WS butterflies of both sexes incur a fitness cost when mated to a non-virgin, while DS butterflies do not. We had predicted that DS but not WS males would change their mating behavior in response to female mating status. However, we found that non-virgin WS males reduced their courtship rates around non-virgin WS females, but that there was no effect of female mating status on either virgin WS male behavior or the behavior of DS males (virgins or non-virgins). We did find DS females to be more likely to mate multiply than WS females, as we had predicted, however DS females were more likely to mate in general, and the effect of a previous mating on likelihood to mate was not greater in WS pairs than in DS pairs. Our results suggest that there may be additional WS-specific selection pressures that may be driving costs of mating with non-virgins in the WS, which we discuss below, in addition to the previously described selection pressures driving different morphologies and courting behaviors for each seasonal form [21, 61]. The WS mating costs coincide with a rearing temperature dependent change in copulatory and pre-copulatory behavior around individuals of the opposite sex with different mating statuses (virgin versus non-virgin). As these individuals were reared in similar social conditions with abundant food, it is unlikely that the observed change in behavioral plasticity is the result of variation in amount of social stimulation or nutrient availability experienced during development, but is instead due to variation in rearing temperature.

The observed temperature dependent changes in mating behavior and cost of mating with a non-virgin suggest that there may be seasonal benefits of being able to detect and respond to mating status in *B*. *anynana*. *B*. *anynana* has not been well studied in nature, so these seasonal benefits cannot be identified definitely at this time. However, survey data suggests that *B*. *anynana* exist at greater densities and that larval and adult food resources are more abundant in wet season conditions relative to dry season conditions [44, 62]. Experimental work also suggests that predation pressures may differ between the wet and dry season, with wet season butterflies experiencing greater predation from insect predators, and dry season butterflies experiencing greater predation from avian predators [46]. Social environment and nutrient availability are known to influence male choosiness and spermatophore content in some species of insects [38, 42], and to play a role in adult neural development and learning ability in a wide range of species [5, 63, 64]. Our data suggest that ambient temperature may also influence neural development and adult behavioral plasticity, particularly in species with alternative morphs (or seasonal polyphenisms). Studies of behavioral plasticity in wild caught *B*. *anynana* butterflies of different seasonal forms would be particularly informative for determining the importance of changes in rearing temperature or other aspects of the natural physical environment to neural development and adult behavioral response to social environment.

Our findings that temperature is sufficient to induce a change in the cost of mating with a non-virgin, and that rearing temperature is sufficient to induce a change in behavioral plasticity, highlights the potential for climate change induced phenotype-environment mismatches in species that use ambient temperature as cues for particular developmental trajectories. Variation in rearing temperature is sufficient to induce both plasticity in adult mating behavior and the broad morphological phenotypic changes necessary to produce either a WS butterfly, adapted to warm, wet season conditions of sub-tropical Africa, or a DS butterfly, adapted to cool, dry season conditions ([21, 44, 48], this study). As environmental conditions shift due to climate change, rearing temperature may become less predictive of adult environment for *B*. *anynana* and other species, and produce animals that are less fit for their environments. This phenotype-environment mismatch in migratory species and species that undergo reproductive diapause has been a cause for concern for some time [6, 7, 9, 11]. However, less attention has been given to the effect of climate change on species with seasonal polyphenisms. Species with seasonal polyphenisms are theorized to have evolved distinct seasonal phenotypes due to distinct selection pressures acting in the different seasons [65], and a reduction in the association between rearing environment and adult environment may be particularly detrimental to these species. Examining the effect of climate change on seasonally polyphenic species may be particularly important in the context of range shifts and expansions, as many species of insects are currently experiencing range shifts (reviewed in [11]), and a shift to novel environments with familiar temperature regimes has the potential to enhance a phenotype-environment mismatch, particularly in systems whose adult phenotypes are highly dependent upon developmental environment.

Here we specifically tested the effect of rearing temperature on behavioral response to different social situations, we did not test whether there was also an effect of rearing temperature on learning ability. Previous work in other systems has shown that deprivation of social stimuli and nutrients during development reduces both learning ability and adult response to multiple social cues [5, 64, 66–68]. In addition, food source and type of social stimuli experienced during development and juvenile periods are known to alter mate selection in multiple insect systems [59, 69–72]. WS *B*. *anynana* butterflies exhibit plasticity in response to mating status (this study), and both sexes also exhibit mate preference learning [59, 72]. It is currently unknown whether DS *B*. *anynana* butterflies also exhibit mate preference learning, or whether DS butterflies exhibit less behavioral plasticity than WS butterflies in general. Eye size, facet number, and facet size are rearing temperature dependent for both male and female *B*. *anynana* butterflies [73]. Variation in these eye traits is also correlated with variation in levels of photoreceptor expression [73, 74]. Thus this developmental plasticity in eye morphology and physiology suggests that adult visual processing ability may also be rearing temperature dependent [73, 74]. It is unclear what other aspects of sensory processing are influenced by rearing temperature, but our data, coupled with that of previous work, suggests that rearing temperature has a significant impact on multiple aspects of neural development in this butterfly.

The behavioral response of both sexes to changes in mating status of the opposite sex suggests that there may be mating status dependent signals in both sexes in *B*. *anynana* that are perceived by the opposite sex. While there is ample evidence that female cuticular hydrocarbon profiles change after copulation in insects, either as a direct result of male manipulation or as a result of a change in diet (reviewed in [75]), this has not yet been tested in *B*. *anynana*. There is also little data on whether mating changes male chemical profiles. However, in species where there is a fitness cost associated with mating with a previously mated male, such as shown here for WS *B*. *anynana*, and in other studies for *Pieris napi*, and *Drosophila melanogaster* [76, 77], it is advantageous for females to be able to detect male mating status. Future research should, thus, try to determine the nature of male signals that may change with sexual experience.

Our data suggests that the reluctance to remate in females and the mating induced reduction in WS female courting rate is independent of spermatophore content. Instead, our results suggest that there is an effect of developmental temperature on some aspect of female physiology that changes in response to copulation in WS females only. The WS female increased reluctance to remate may have evolved in response to a lower quality WS first spermatophore relative to a DS spermatophore [21], or due to the presence of substances that negatively impact female longevity in WS male spermatophores. Male spermatophore quality and the corresponding sex role reversal in *B*. *anynana* appears to be induced by rearing temperature instead of nutrient availability; the latter driving sex role reversal and male investment in Orthoperans [21, 38, 42]. It is, however, unclear whether there are rearing temperature dependent effects of spermatophores on female attractiveness, as WS mated males and not WS virgin males reduced their courting rates around non-virgin females, and mated males were not used for the spermatophore comparison assays. The presence of spermicides in butterfly spermatophores is currently unknown, but *Drosophila* seminal fluids are known to be toxic to females [78], and there is some evidence in other butterfly species that mating may be toxic to females [79]. While male spermatophore content is responsible for inducing reluctance to copulate in females and reducing female attractiveness in a variety of insect species, additional factors appear to be in play in *B*. *anynana*. Future research examining the physiological and biochemical changes associated with copulation in WS and DS females, and in WS males, will greatly enhance our understanding of the multiple factors influencing female post-copulatory behavior, as well as the male’s longer second copulation, in response to developmental temperature.

Our data show that DS individuals exhibit fewer changes in behavior with changes in mating status than WS individuals, and that mating with a previously mated individual does not negatively impact DS female or male longevity as it impacts WS female and male longevity. These results did not fit our original hypotheses that DS females would benefit more from mating multiply, and DS males would incur a greater cost, relative to WS individuals. In addition, we did not observe that DS females increase, and males reduce, their longevity with the first mating, as had been show in a previous study [21]. This discrepancy may be the result of DS butterfly behavior and reproductive costs being influenced by other abiotic factors in addition to temperature, such as relative humidity or nutrient availability. This hypothesis is supported by the fact that butterflies in our study, which did not exhibit DS female specific mating benefits, or DS male specific mating costs, were fed for three days after emergence, while adult females and males in the previous study, which did show DS female specific mating benefits and DS male specific mating costs, were not given any food after emergence [21]. Nutrient availability may heighten the effect of spermatophore quality on female longevity, and spermatophore loss on male longevity, in this system. Future work should determine whether other developmental environmental factors, such as relative humidity or nutrient availability, are also used as cues for behavioral plasticity in this (and other) seasonally polyphenic species.

Our experimental design also differed from Prudic et al. in that lifespan was observed in the respective rearing temperatures in order to produce as natural ambient temperature conditions as possible in the laboratory setting, instead of measuring lifespan at a common temperature (25°C) [21]. Both our and Prudic et al.’s results, however, suggest that DS females may gain from multiple matings, especially in the absence of any adult food resource, and, therefore, DS females do not alter their courtship behavior upon a first mating, whereas WS females do. The use of rearing environment to set the degree of plasticity that an adult exhibits in mating interactions, and thus to restrict the set of adult behaviors performed upon emergence, may allow butterflies to save energy in environments where nutrient sources are scarce (such as the DS for *B*. *anynana*), as behavioral plasticity and learning ability are energetically costly [2]. Given the energetic costs of behavioral plasticity and learning ability, climate change induced decoupling of rearing temperature and seasonal environment, such as warmer temperatures being associated with drier environments containing less food and foliage coverage, may prove detrimental to species such as *B*. *anynana*, which use rearing temperature as a cue for determining adult morphological and behavioral phenotypes. However, the increase in adult behavioral plasticity induced by warm rearing temperatures may also prove to be beneficial to butterflies experiencing novel abiotic and social environments, as it may facilitate behavioral modifications that allow these butterflies to adjust to a wide range of environments. Future work should explore the association between rearing temperature, neural development, and behavioral plasticity in multiple systems to determine whether there may be unexpected effects of climate change on the ability of animals to modify their behavior in response to changes in their environment.

## Conclusions

We demonstrate that rearing temperature influences the direct cost of mating with a previously mated individual instead of a virgin; and that rearing temperature also influences adult male and female mating behavior plasticity in response to own mating status and the mating status of a potential mate. Our finding that mating behavior plasticity is mediated by rearing temperature suggests that brain development and neuroplasticity may be influenced by rearing temperature in *Bicyclus anynana*. Future research should explore the pervasiveness of this phenomenon across taxa, as changing global ambient temperatures may have the potential to influence the ability of multiple species of animals to respond to novel or changing environments.

## Supporting Information

S1 Table Parametric Survival Model of *B*. *anynana* lifespan. (PDF)

S2 Table Correlations of male and female activity levels by treatment. (PDF)

S3 Table GLM effect tests for factors influencing copulation rates. (PDF)

S4 Table GLM of best fit for copulation duration, fit of all models tested. (PDF)

S5 Table GLM effect tests for factors influencing male and female activity, all data pooled. (PDF)

S6 Table GLM effect tests for factors influencing WS male and female activity. (PDF)

S7 Table GLM effect tests for factors influencing DS male and female activity. (PDF)
