# Supplementary material for: Rearing Temperature Influences Adult Response to Changes in Mating Status
Source: PLoS One. 2016 Feb 10;11(2):e0146546. doi: 10.1371/journal.pone.0146546 (PMC4749170; doi:10.1371/journal.pone.0146546)
Supplement: S1 Table — (PDF) [file pone.0146546.s001.pdf]

S1 Table Parametric Survival Model of *B. anynana* lifespan.

	χ^2	p-value
Whole model	**96.667**	**<0.0001**
Factors		
Sex	**6.337**	**0.012**
Season	**14.613**	**0.0001**
Sex * Season	0.064	0.800
Number of matings	**7.136**	**0.008**
Sex * Number of matings	0.315	0.575
Season * Number of matings	0.639	0.424
Sex * Season * Number of matings	0.300	0.584
Mating status of mate	**18.772**	**<0.0001**
Sex * Mating status of mate	1.643	0.440
Season * Mating status of mate	**7.477**	**0.024**
Sex * Season * Mating status of mate	0.007	0.997
Number of mates * Mating status of mates	**14.148**	**0.0002**
Sex * Number of mates * Mating status of mates	0.123	0.725
Season * Number of Mates * Mating status of mates	0.971	0.324
Sex * Season * Number of mates * Mating status of mates	0.053	0.817

Significant factors are in bold.
