# Supplementary material for: Rearing Temperature Influences Adult Response to Changes in Mating Status
Source: PLoS One. 2016 Feb 10;11(2):e0146546. doi: 10.1371/journal.pone.0146546 (PMC4749170; doi:10.1371/journal.pone.0146546)
Supplement: S2 Table — (PDF) [file pone.0146546.s002.pdf]

S2 Table Correlations of male and female activity levels by treatment.

	**WS**			**DS**		
Treatment	Slope	R^2	p-value	slope	R^2	p-value
**PC 1**						
VM x VF	**0.830**	**0.296**	**0.044**	0.252	0.088	0.304
VM x MF	**1.365**	**0.521**	**0.004**	**0.316**	**0.320**	**0.028**
MM x VF	0.127	0.136	0.159	**1.082**	**0.487**	**0.004**
MM x MF	0.728	0.203	0.091	0.608	0.206	0.103
**PC 2**						
VM x VF	**0.338**	**0.364**	**0.022**	-0.057	0.004	0.834
VM x MF	-0.529	0.268	0.058	-0.003	0.003	0.836
MM x VF	-0.121	0.113	0.203	-0.476	0.202	0.093
MM x MF	1.697	0.078	0.313	-0.496	0.045	0.467
**PC 3**						
VM x VF	-0.368	0.023	0.608	-0.188	0.052	0.432
VM x MF	0.443	0.046	0.462	0.095	0.018	0.635
MM x VF	-0.326	0.194	0.088	-0.297	0.029	0.543
MM x MF	-0.164	0.001	0.904	**-1.92**	**0.508**	**0.004**

Values in bold have a p-value <0.05. VM= virgin male, VF= virgin female, MM= mated male, MF= mated female.
