# Supplementary material for: Rearing Temperature Influences Adult Response to Changes in Mating Status
Source: PLoS One. 2016 Feb 10;11(2):e0146546. doi: 10.1371/journal.pone.0146546 (PMC4749170; doi:10.1371/journal.pone.0146546)
Supplement: S3 Table — (PDF) [file pone.0146546.s003.pdf]

S3 Table GLM effect tests for factors influencing copulation rates.

	**Frequency of Copulation**		**Latency to Copulation**	
**Factor**	χ^2	p-value	χ^2	p-value
Rearing environment	**24.673**	**<0.0001**	**22.854**	**<0.0001**
Female mating status	**24.673**	**<0.0001**	**22.327**	**<0.0001**
Male mating status	0.021	0.8859	0.000	0.991
Rearing Env. * Female m. status	0.044	0.8340	0.009	0.922
Rearing Env. * Male m. status	0.6721	0.4141	0.574	0.449
F. mating status * M. mating status	0.2825	0.5959	0.127	0.721
Rearing Env. * Female m. status * Male. status	1.0902	0.2982	0.697	0.404

Significant effects are in bold.
