# Supplementary material for: Rearing Temperature Influences Adult Response to Changes in Mating Status
Source: PLoS One. 2016 Feb 10;11(2):e0146546. doi: 10.1371/journal.pone.0146546 (PMC4749170; doi:10.1371/journal.pone.0146546)
Supplement: S4 Table — (PDF) [file pone.0146546.s004.pdf]

S4 Table GLM of best fit for copulation duration, fit of all models tested.

**Model: factors**	AICc	χ^2	p-value
**Model 1**: Season, male mating status, season * male mating status	437.45	9.597	0.022
**Model 2:** Male mating status	438.829	3.427	0.041
**Model 3:** Female mating status	439.94	2.313	0.128
**Model 4**: Season, female mating status, male mating status	440.25	6.794	0.079
**Model 5:** Season	441.79	0.465	0.495
**Model 6**: Female mating status, male mating status, female mating status * male mating status	441.92	5.128	0.163
**Model 7**: Season, female mating status, season * female mating status	442.33	4.7071	0.194
**Model 8**: Full factorial containing Season, Male mating status, Female mating status	443.58	11.456	0.0753
