# Supplementary material for: Rearing Temperature Influences Adult Response to Changes in Mating Status
Source: PLoS One. 2016 Feb 10;11(2):e0146546. doi: 10.1371/journal.pone.0146546 (PMC4749170; doi:10.1371/journal.pone.0146546)
Supplement: S5 Table — (PDF) [file pone.0146546.s005.pdf]

S5 Table GLM effect tests for factors influencing male and female activity, all data pooled.

	**Female**							
	Courting		PC1		PC2		PC3	
	χ^2	p-value	χ^2	p-value	χ^2	p-value	χ^2	p-value
Whole model	9.385	0.226	10.587	0.158	13.020	0.072	**17.161**	**0.016**
Factors								
Season	1.028	0.311	2.573	0.109	0.492	0.483	**12.984**	**0.0003**
Female mating status	2.080	0.149	1.644	0.120	1.705	0.192	0.839	0.360
Male mating status	0.331	0.565	1.263	0.261	4.193	0.040	0.012	0.914
Season * Female m. status	2.319	0.128	0.239	0.625	2.282	0.131	0.283	0.595
Season * Male m. status	2.615	0.106	1.143	0.285	1.967	0.161	0.191	0.662
F. mating status * M. mating status	1.532	0.216	1.798	0.180	3.071	0.080	0.015	0.904
Season * F. mating status * M. mating status	0.268	0.605	0.507	0.477	0.303	0.5821	0.026	0.871
	**Male**							
	Courting		PC1		PC2		PC3	
	χ^2	p-value	χ^2	p-value	χ^2	p-value	χ^2	p-value
Whole model	11.327	0.125	**27.570**	**0.0003**	5.212	0.634	7.425	0.386
Factors								
Season	4.824	0.028	**12.932**	**0.0003**	0.197	0.657	0.015	0.902
Female mating status	2.004	0.157	0.001	0.938	0.126	0.722	0.038	0.846
Male mating status	1.046	0.306	2.657	0.103	0.296	0.586	2.864	0.091
Season * Female m. status	2.048	0.152	1.410	0.235	1.567	0.211	0.038	0.847
Season * Male m. status	0.391	0.532	1.668	0.196	0.342	0.558	1.491	0.222
F. mating status * M. mating status	3.446	0.063	2.440	0.118	1.306	0.253	0.287	0.592
Season X F. mating status * M. mating status	0.119	0.730	0.094	0.759	1.708	0.191	1.929	0.165

Significant factors are in bold.
