# Supplementary material for: Rearing Temperature Influences Adult Response to Changes in Mating Status
Source: PLoS One. 2016 Feb 10;11(2):e0146546. doi: 10.1371/journal.pone.0146546 (PMC4749170; doi:10.1371/journal.pone.0146546)
Supplement: S6 Table — (PDF) [file pone.0146546.s006.pdf]

S6 Table GLM effect tests for factors influencing WS male and female activity

	**WS Female**							
	Courting		PC1		PC2		PC3	
	χ^2	p-value	χ^2	p-value	χ^2	p-value	χ^2	p-value
Whole model	**8.856**	**0.031**	4.479	0.214	**12.003**	**0.007**	0.221	0.974
Factor								
Female mating status	**5.393**	**0.020**	1.292	0.256	**4.272**	**0.039**	0.067	0.796
Male mating status	2.996	0.083	1.970	0.160	**6.347**	**0.012**	0.133	0.715
F. mating status * M. mating status	1.929	0.165	0.165	0.684	2.887	0.089	0.036	0.849
	**WS Male**							
	Courting		PC1		PC2		PC3	
	χ^2	p-value	χ^2	p-value	χ^2	p-value	χ^2	p-value
Whole model	5.609	0.132	3.064	0.382	**9.666**	**0.022**	5.356	0.147
Factor								
Female mating status	0.001	0.980	1.425	0.233	1.078	0.299	0.218	0.641
Male mating status	0.389	0.533	0.136	0.712	1.694	0.193	0.329	0.566
F. mating status * M. mating status	5.488	0.019	1.827	0.176	**7.683**	**0.006**	5.217	0.022

Significant factors are in bold.
