# Supplementary material for: Rearing Temperature Influences Adult Response to Changes in Mating Status
Source: PLoS One. 2016 Feb 10;11(2):e0146546. doi: 10.1371/journal.pone.0146546 (PMC4749170; doi:10.1371/journal.pone.0146546)
Supplement: S7 Table — (PDF) [file pone.0146546.s007.pdf]

S7 Table GLM effect tests for factors influencing DS male and female activity

	DS Female							
	Courting		PC1		PC2		PC3	
	χ^2	p-value	χ^2	p-value	χ^2	p-value	χ^2	p-value
Whole model	0.602	0.896	3.707	0.295	1.117	0.773	1.408	0.704
Factors								
Female mating status	0.003	0.958	0.402	0.526	0.019	0.889	1.169	0.280
Male mating status	0.452	0.501	0.002	0.965	0.196	0.658	0.061	0.805
F. mating status * M. mating status	0.216	0.643	2.641	0.105	0.668	0.414	0.000	0.975
	**DS Male**							
	Courting		PC1		PC2		PC3	
	χ^2	p-value	χ^2	p-value	χ^2	p-value	χ^2	p-value
Whole model	3.808	0.283	5.593	0.133	0.894	0.827	3.071	0.509
Factors								
Female mating status	2.250	0.134	0.513	0.474	0.797	0.372	0.000	0.999
Male mating status	0.758	0.384	2.703	0.100	0.001	0.982	2.553	0.110
F. mating status * M. mating status	1.358	0.244	1.118	0.290	0.008	0.927	0.223	0.637

Significant effects are in bold.
